# A Role for Borg5 During Trophectoderm Differentiation

**DOI:** 10.1002/stem.428

**Published:** 2010-04-15

**Authors:** Queenie P Vong, Zhonghua Liu, Jae Gyu Yoo, Rong Chen, Wen Xie, Alexei A Sharov, Chen-Ming Fan, Chengyu Liu, Minoru SH Ko, Yixian Zheng

**Affiliations:** 1 Department of Embryology, Carnegie Institution for ScienceBaltimore, Maryland, USA; 2 Howard Hughes Medical InstituteBaltimore, Maryland, USA; 3 Developmental Genomics and Aging Section, Laboratory of Genetics, National Institute on AgingNIH, Baltimore, Maryland, USA; 4 Transgenic Core Facility, National Heart, Lung, and Blood InstituteNIH, Bethesda, Maryland, USA

**Keywords:** Borg5, Cdx2, Embryonic stem cells, Trophectoderm, Cell morphogenesis, Transcription, Differentiation

## Abstract

Stem cell differentiation is accompanied by a gradual cellular morphogenesis and transcriptional changes. Identification of morphological regulators that control cell behavior during differentiation could shed light on how cell morphogenesis is coupled to transcriptional changes during development. By analyzing cellular behavior during differentiation of mouse embryonic stem cells (ESCs), we uncover a role of Borg5 (binder of Rho guanosine 5′-triphosphatase 5) in regulating trophectoderm (TE) cell morphogenesis. We report that differentiation of ESCs toward TE is accompanied by enhanced actin protrusion and cell motility that require upregulation of Borg5. Borg5 interacts with both Cdc42 and atypical protein kinase C (aPKC) and functions downstream of Cdc42 to enhance TE cell motility. Borg5 is required for the sorting of differentiating TE to the outside of ESCs in vitro. In developing embryos, Borg5 protein localizes to cell–cell contacts and the cytoplasm after compaction. It exhibits higher levels of expression in outer cells than in inner cells in morula and blastocysts. Reduction of Borg5 disrupts aPKC localization and inhibits blastocyst formation. Since Cdx2 and Borg5 facilitate each other's expression as ESCs differentiate toward TE, we propose that cell morphogenesis is coupled with transcriptional changes to regulate TE differentiation. Our studies also demonstrate the utility of ESCs in identifying morphological regulators important for development.

## INTRODUCTION

Lineage segregation during development and stem cell differentiation depends on transcriptional regulation and is accompanied by cell morphological changes. Much is known about how transcriptional programs regulate cell differentiation and how general cell morphological regulators control cell motility, polarity, and adhesion during development. By contrast, little is known about how lineage specific transcriptional regulation is coupled to correspondingly unique cell morphological changes during the building of tissues and organs. Understanding this coupling at the cellular level is important because it would help to define the steps that stem and progenitor cells take to differentiate.

Embryonic stem cells (ESCs) are derived from the inner cell mass (ICM) of preimplantation mammalian blastocysts [1], which contains two populations of cells, the ICM and the trophectoderm (TE) [2]. The segregation of TE from ICM becomes apparent by the blastocyst stage when the TE encloses the blastocoel cavity and the ICM. Expression of transcription factors such as Oct3/4 (also called Oct4 or Pou5f1), Sox2, and Nanog is restricted to the ICM in mouse embryos [3]. These transcription factors are also required for maintaining the pluripotency of ESC in vitro. By contrast, expression of Cdx2, which is restricted to the outer cells in blastocysts, is essential for the development of the TE lineage [4–9].

ESCs can be induced to differentiate into different cell lineages in vitro and they have been used extensively to study transcriptional changes during differentiation [10]. Here, we show that ESCs can also be used to study cell behaviors during differentiation into specific lineages. We report the identification of Borg5 as a morphological regulator for TE differentiation using ESCs as a model system.

## MATERIALS AND METHODS

All ESCs were cultured in the absence of feeder cells (except when specifically indicated) on gelatin-coated dishes. To image ESC differentiation in a LiveCell™ Chamber (Pathology Devices, Westminster, MD, USA), time-lapse recording at multiple positions per well were acquired on an inverted microscope (TE2000-U, Nikon, Melville, NY, USA) controlled by IPLab4.0 every 5 minutes for 2 days. Closed-loop feedback from a Z Encoder Probe (Prior Scientific, Rockland, MA, USA) was used to control focus drifts. For additional detailed materials and methods, please refer to the Supporting Information.

## RESULTS

### Unique Cell Behaviors Accompany ESC Differentiation Toward Specific Lineages

We used RNA interference (RNAi) to downregulate *Oct3/4*, *Sox2*, or *Nanog* [11] in feeder-free E14 ESCs to study cellular changes during early differentiation toward different lineages. Reduction of *Oct3/4* or *Nanog* in ESCs has been shown to cause differentiation toward TE [6] or primitive endoderm [12, 13], respectively. Reduction of *Sox2* by RNAi leads to multilineage differentiation including TE [11]. However, knockdown of *Sox2* in ESCs in which *Sox2* is expressed from a tetracycline (Tc)-controlled transgene leads to mostly TE differentiation [14]. The difference in differentiation after *Sox2* downregulation in the two studies may be caused by different efficiencies of *Sox2* downregulation.

One day after RNAi treatment, each gene was reduced (Supporting Information Fig. S1A). Daily inspection of cells showed that by day 6 all three RNAi caused formation of differentiated flat cells (Supporting Information Fig. S1B, C). To analyze the early phase of cellular behavior during differentiation, we carried out time-lapse imaging of differentiating ESCs within the first 2 days of RNAi. Although control ESC colonies exhibited short and dynamic cellular protrusions as the colonies expanded through cell division (Supporting Information Movie 1), *Oct3/4* RNAi caused individual ESCs in the colony to send out long cell processes with cell clusters and colonies migrated toward each other (Supporting Information Movie 2). *Nanog* RNAi-treated ESCs flattened into uniform cuboidal-shaped cells with multiple short and dynamic cellular processes (Supporting Information Movie 3), whereas individual *Sox2* RNAi-treated ESCs exhibited various morphology and migratory behavior consistent with its differentiation toward different lineages (Supporting Information Movie 4). Therefore, distinct cellular behaviors accompany the differentiation of ESCs into unique lineages.

Next, we characterized cell behaviors more quantitatively. Since it is difficult to track individual cell motility by phase contrast microscopy as ESCs differentiate, we used the displacement of histone-green fluorescent protein (GFP) labeled nuclei to measure cell movement during differentiation. We created E14 ESCs expressing histone 2B-GFP, E14-H2B-GFP. These cells have the same morphology as the parental E14 ESCs and are capable of generating germline transmission (Supporting Information Fig. S2). For easy tracking of individual GFP positive nuclei by time-lapse microscopy, E14-H2B-GFP ESCs were spiked into unlabeled E14 ESCs (Supporting Information Fig. S1D). The distance a nucleus moved before nuclear envelope breakdown (NEBD) was measured as D1. After nuclear division, distances between the NEBD mother nucleus and the two daughter nuclei were measured as D2 and D3 (Supporting Information Fig. S1D, E and Movie 5). The sum of D1, D2, and D3 allowed us to assess the degree of cell migration. We found that reduction of Oct3/4 resulted in the strongest enhancement of cell motility followed by Nanog reduction, whereas Sox2 reduction did not cause a significant overall increase in cell motility (Supporting Information Fig. S1F,G).

**Figure 1:**
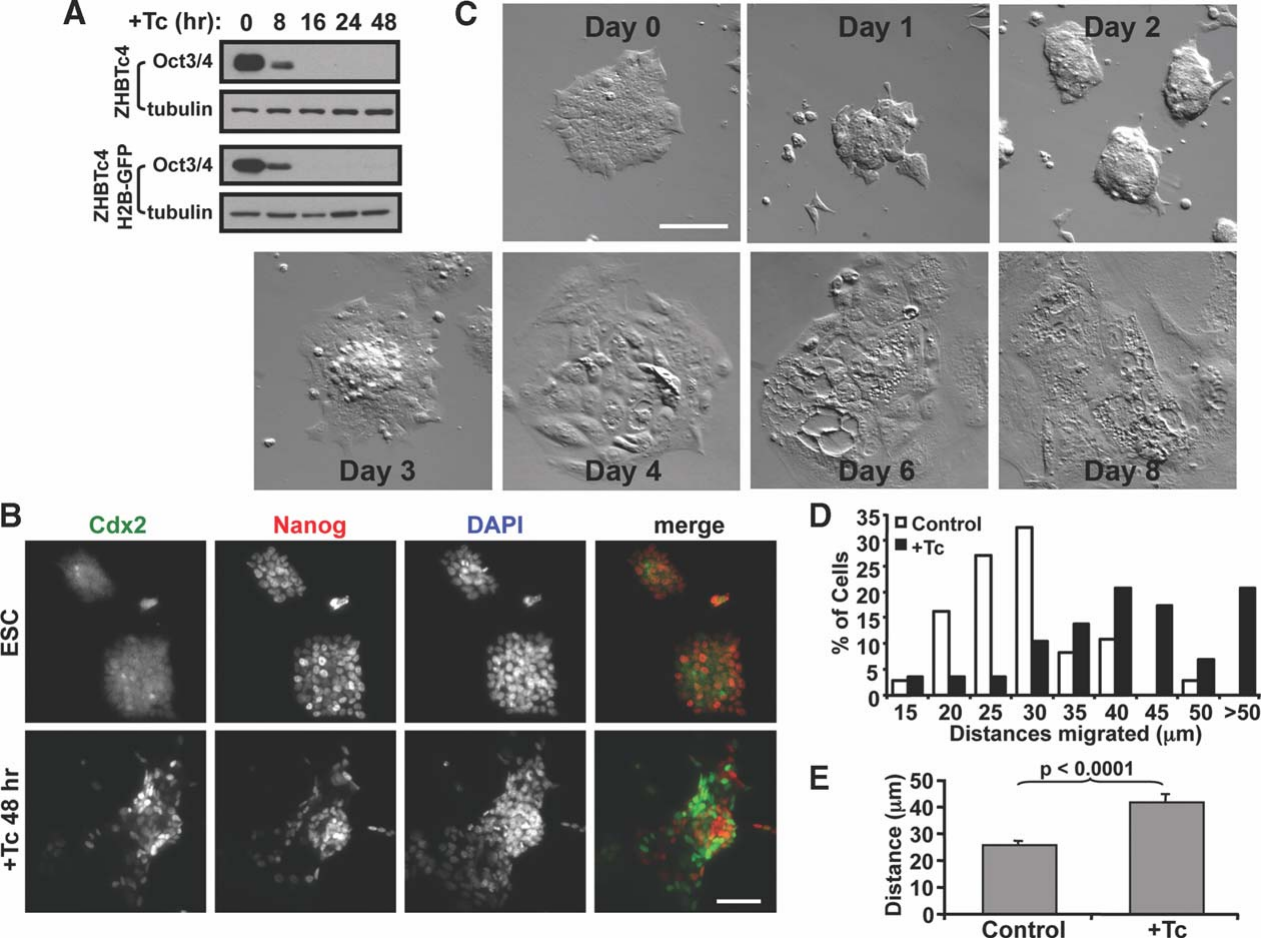
Differentiation of ZHBTc4 ESCs after Tc addition. (A): Addition of Tc induces rapid downregulation of Oct4 in both ZHBTc4 ESCs and ZHBTc4-H2B-green fluorescent protein ESCs. (B): Cdx2 protein appeared in the nuclei of differentiating ZHBTc4 ESCs 48 hours after Tc addition. At this time point many cells still expressed Nanog. Scale bar, 100 μm. (C): Tc addition induces distinct morphological changes in ZHBTc4 ESCs. Tc was added to the cells cultured in ES medium. Images were taken each day after Tc addition up to 8 days. Scale bar, 100 μm. (D, E): Reduction of Oct3/4 by Tc addition caused a significant increase in cell migration. Shown are distribution of distances migrated for >30 cells in each condition (D) and average distances migrated (E). Error bars, SEM. *p* value was calculated using Student's *t* test. Abbreviations: DAPI, 4′,6-diamidino-2-phenylindole; ESC, embryonic stem cell; Tc, tetracycline.

The above data suggest that specific regulators of cell morphogenesis might be upregulated very early to mediate unique cellular behaviors as ESCs differentiate into a specific lineage. Since cell motility is most pronounced during TE differentiation, we chose to focus on identifying TE specific morphological regulators responsible for TE cell motility using microarray analysis. We turned to the engineered ESCs, ZHBTc4, in which the pluripotency is maintained by a Tc-regulated *Oct3/4* transgene [15], therefore TE differentiation can be induced more efficiently and homogeneously by Tc addition. Tc addition caused efficient reduction of Oct3/4 (Fig. 1A) and the appearance of lineage specific transcription factor Cdx2 (Fig. 1B). Consistent with RNAi of *Oct3/4* in E14 ESCs, Tc addition caused enhanced migration of cell colonies toward each other followed by cell flattening (Fig. 1C–1E).

### Identification of Borg5 That Is Upregulated in Differentiating TE Cells

To identify genes that are upregulated early during TE differentiation, we carried out microarray analyses 12 hours after Tc addition and found that 13 genes were upregulated by at least twofold [16, 17]. Among these, only two genes, *Cdx2* and *Borg5*, were upregulated by >10-fold (Supporting Information Table 1). *Borg5* [binder of Rho guanosine 5′-triphosphatase (GTPase) 5], which has also been referred to as *Cdc42EP1* (Cdc42 effector protein 1), encodes a protein that binds to RhoGTPases. Since RhoGTPases bind to various effector proteins to regulate cell morphogenesis, Borg5 is likely a morphological regulator that could influence TE specific cell morphogenesis and transcription during differentiation.

**Figure 2:**
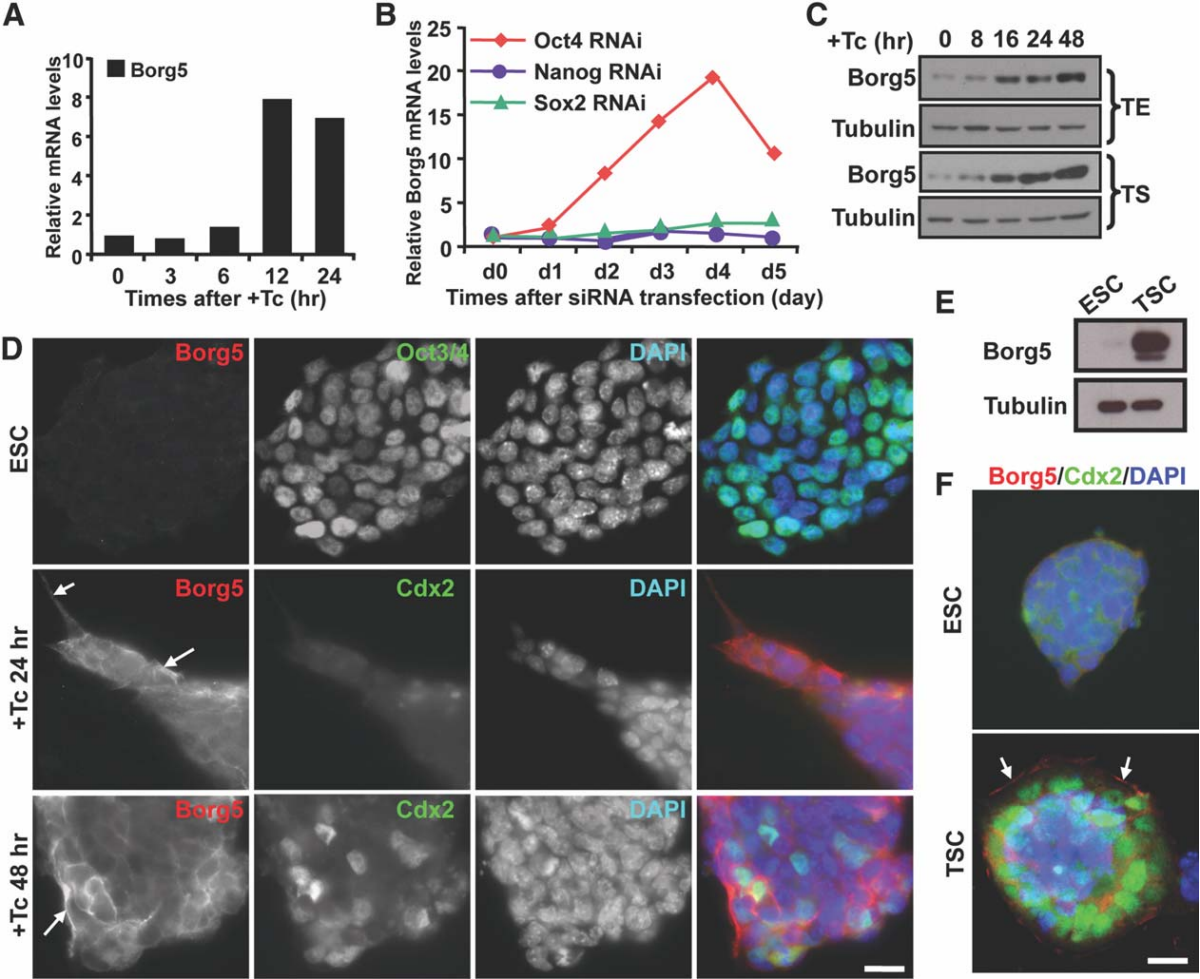
Expression of Borg5 after Oct3/4 downregulation. (A):*Borg5* mRNA was upregulated in ZHBTc4 cells 12 hours after Tc addition as assayed by quantitative real-time polymerase chain reaction. (B):*Borg5* mRNA level was upregulated after *Oct3/4* siRNA treatment but not after *Nanog* or *Sox2* siRNA treatments in E14 cells. The delayed Borg5 mRNA upregulation when compared with Tc addition in ZHBTc4 cells may reflect the slower and heterogeneous reduction of *Oct3/4* mediated by siRNA. (C): Borg5 protein was upregulated 16 hours after Tc addition in both TE and TS culturing conditions based on Western blotting analysis. (D): Borg5 protein localization in ZHBTc4 ESCs and the differentiating ZHBTc4 cells 24 and 48 hours after Tc addition based on immunofluorescence staining. Oct3/4 and Cdx2 antibodies were used to label the undifferentiated and differentiating ESCs, respectively. Short and long arrows point to Borg5 in the cell process and along the edges of cells, respectively. (E): Borg5 protein is highly expressed in blastocyst-derived TSCs. (F): Borg5 protein localization in ESCs and TSCs. Merged images of Borg5 (red), Cdx2 (green), and DAPI (blue) are shown. Arrows point to Borg5 concentrated along the cell periphery of the TSC colony. Scale bars, 20 μm. Abbreviations: DAPI, 4′,6-diamidino-2-phenylindole; ESC, embryonic stem cell; RNAi, RNA interference; siRNA, small interfering RNA; Tc, tetracycline; TE, trophectoderm; TS, trophoblast stem cell; TSC, trophoblast stem cell.

Using quantitative real-time polymerase chain reaction (qRT-PCR), we characterized the expression of *Borg5* and found that *Borg5* mRNA is strongly upregulated after reduction of Oct3/4 in both ZHBTc4 (Fig. 2A) and E14 ESCs (Fig. 2B), but not after reduction of Nanog or Sox2 (Fig. 2B). Therefore, *Borg5* transcription is suppressed in ESCs and becomes upregulated specifically during TE differentiation. We analyzed previous whole genome ChIP-Seq studies of Oct3/4 binding sites in ESCs [18, 19] and found that there is no Oct3/4 binding site within 30 kb upstream and downstream of the transcriptional start site (TSS) of *Borg5*. Using ChIP-qPCR, we did not detect any Oct4 binding within 2 kb upstream of *Borg5* TSS (data not shown). Thus, Oct3/4 is likely to indirectly suppress *Borg5* expression in ESCs.

Borg5 protein is expressed at low levels in ESCs, but becomes upregulated shortly after Oct3/4 reduction in both ZHBTc4 (Fig. 2C) and E14 cells (Supporting Information Fig. S3) either in ESC medium (favoring terminal differentiation of TE) or in trophoblast stem cell (TSC) medium (allowing derivation of TSC). Immunofluorescence staining revealed that Borg5 is localized to the cytoplasm and often concentrated at the cell–cell junctions and along the edges of differentiating colonies (Fig. 2D). Borg5 is also expressed at higher levels in the blastocyst-derived TSCs [6] than ESCs (Fig. 2E, 2F). Thus, Borg5 expression is enhanced in the TE lineage during ESC differentiation.

### Borg5 Regulates Actin Protrusion and Cell Motility During TE Differentiation from ESCs Downstream of Cdc42

**Figure 3:**
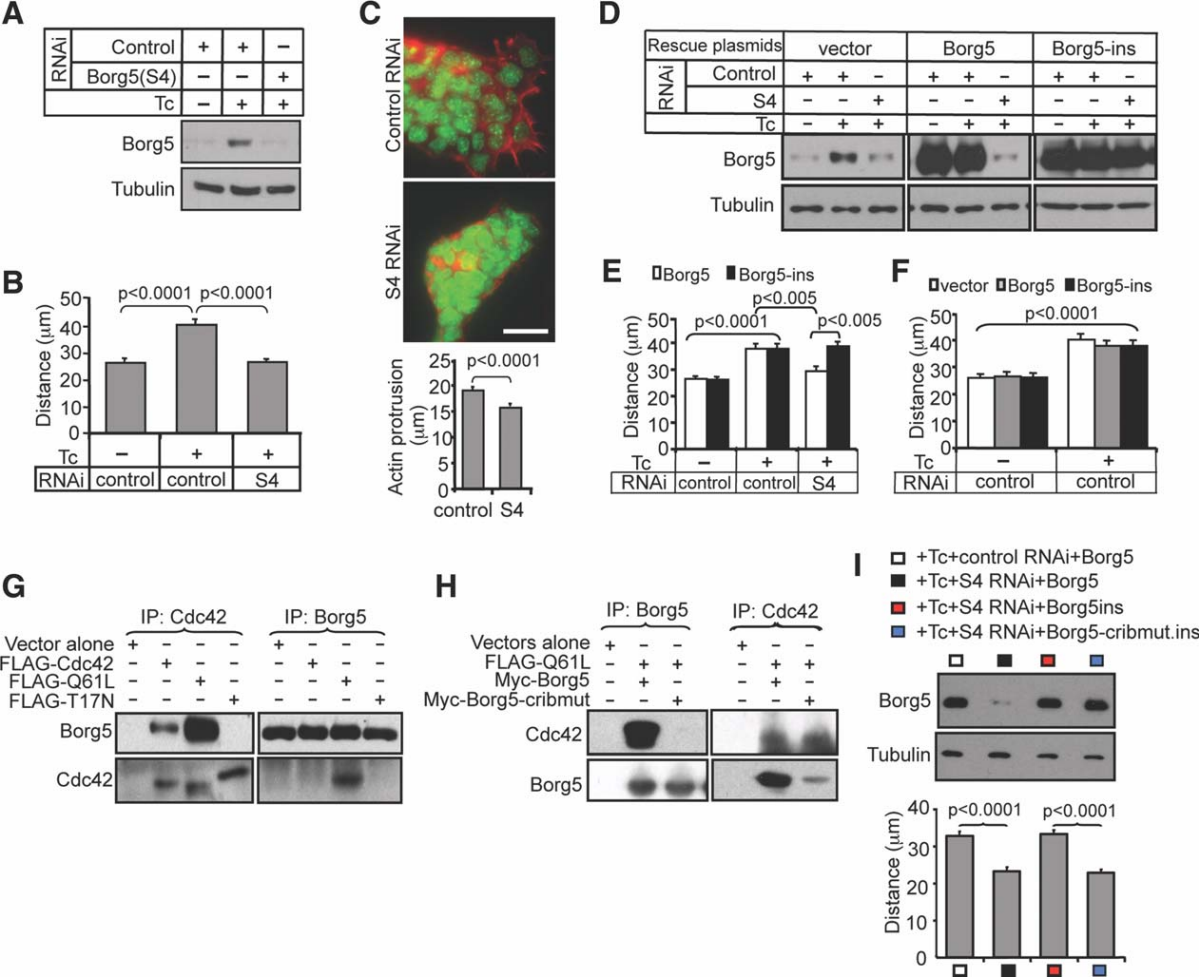
Borg5 regulates the motility of differentiating trophectoderm (TE) cells. (A):*Borg5* small interfering RNA (siRNA) (S4 oligo) successfully suppressed Borg5 upregulation after addition of Tc. (B): Borg5 reduction by RNAi significantly inhibited cell migration induced by Tc. (C): Borg5 promotes actin protrusions in differentiating TE cells. Actin (red) and nuclei (green) were labeled with phalloidin and DAPI, 4′,6-diamidino-2-phenylindole respectively. Scale bar, 20 μm. The length of actin protrusion was measured from the centroid of each nucleus at the periphery of colonies to the tip of the longest actin protrusion from that cell at 48 hours after Tc addition. (D): Expressing Borg5 and Borg5-ins (insensitive to Borg5 S4 siRNA oligo) cDNA in ZHBTc4 cells in the presence or absence of Tc and S4 oligo as judged by Western blotting analysis. Borg5-ins, but not Borg5, cDNA could rescue Borg5 protein expression in the presence of S4 oligo. Borg5 protein was overexpressed to similar levels from Borg5 cDNA (in the absence of S4 oligo) or Borg5-ins cDNA (in the presence of S4 oligo) in the presence or absence of Tc. But this overexpression did not cause additional increase in cell motility (F). (E): Expression of Borg5-ins restored cell migration induced by Tc addition in the presence of S4 siRNA. (F): Elevated Borg5 expression in embryonic stem cells (ESCs) (−Tc) or in differentiating ESCs (+Tc) did not induce additional cell motility when compared with cells (vector controls) that did not have elevated Borg5. Therefore, overexpressing Borg5 did not cause a gain of function in cell motility. (G): Borg5 interacts with guanosine triphosphate (GTP)-bound Cdc42. FLAG-tagged Cdc42 wild-type (FLAG-Cdc42), GTP-bound Cdc42Q61L (FLAG-Q61L), or guanosine diphosphate-bound Cdc42T17N (FLAG-T17N) was expressed in the differentiating TE cells. Immunoprecipitations were carried out using Borg5 or FLAG antibodies. (H): Borg5 interacts with GTP-bound Cdc42 through its Cdc42/Rac interactive binding (CRIB) motif. FLAG-tagged forms of Cdc42 and Myc-tagged wild-type Borg5 (Myc-Borg5) or Myc-Borg5 with point mutations in the CRIB motif (Myc-Borg5-cribmut) were expressed. Immunoprecipitations using either immunoprecipitations using either Myc or FLAG antibodies or FLAG antibodies showed that the interaction between Cdc42Q61L and Borg5 is mediated by the CRIB motif of Borg5. (I): The CRIB motif of Borg5 is required for TE cell motility. Western-blotting analyses of Borg5 expression (wild-type Borg5 or Borg5-cribmut) in ZHBTc4 cells that were differentiating toward TE (+Tc) and treated with either control or *Borg5* small interfering RNA. Expression of the RNAi-insensitive Borg5 containing mutations in the CRIB motif failed to rescue cell motility in the differentiating TE treated with Borg5 siRNA. The differently colored squares indicated the conditions for each treatment. Error bars, SEM. *p* values were calculated using Student's *t* test. Abbreviations: FLAG, DYKDDDDK octapeptide; RNAi, RNA interference; Tc, tetracycline.

We reasoned that as a RhoGTPase binding protein [20–22] Borg5 might regulate actin protrusion and cell motility during TE differentiation from ESCs. Two small interfering RNA (siRNA) oligos, S2 and S4, targeting the 5′-untranslated region and coding region of the gene, respectively, efficiently inhibited Borg5 upregulation and cell motility in ZHBTc4 ESCs as they differentiate toward TE in the presence of Tc (Fig. 3A, 3B, Supporting Information Fig. S4A–C). Reduction of Borg5 also reduced the length of actin protrusions formed at the periphery of differentiating cell colonies when compared with controls (Fig. 3C), suggesting that Borg5 can regulate actin assembly. Expression of *Borg5* cDNA lacking the 5′-untranslated region (Supporting Information Fig. S4A,D, and E) or *Borg5* cDNA carrying silent mutations (Borg5-ins) that render it insensitive to RNAi (Fig. 3D–3F) rescued the cell motility defects caused by S2 or S4 siRNA, respectively. Therefore, upregulation of Borg5 during TE differentiation from ESCs is responsible for TE cell motility. We found that constitutive overexpression of Borg5 using an episomal system in ESCs did not cause increased cell motility or cell differentiation (data not shown). This suggests that Borg5 alone does not drive differentiation and a differentiating TE environment is required for Borg5 to exert its function in regulating cell morphogenesis.

Since Borg5 contains the Cdc42/Rac interactive binding (CRIB) motif, we next asked whether it functions downstream of Cdc42 to regulate cell motility during TE differentiation. We first confirmed that Borg5 interacts with the guanosine triphosphate-bound form of Cdc42 (Cdc42Q61L) (Fig. 3G) [20–22]. We then showed that this interaction was reduced substantially when point mutations were made in the CRIB motif (Fig. 3H). To test whether the CRIB motif is required for cell motility, we created a CRIB mutant of Borg5, Borg5-cribmut-ins, which is insensitive to the S4 RNAi. Borg5-cribmut-ins, like Borg5-ins, is expressed in the presence of siRNA (Fig. 3I). However, Borg5 CRIB mutant failed to rescue cell motility during TE differentiation (Fig. 3I). Therefore, Borg5 regulates cell motility as an effector protein of RhoGTPases during ESC differentiation toward the TE lineage.

### Borg5 and Cdx2 Facilitate Each Other's Expression During TE Differentiation from ESCs

**Figure 4:**
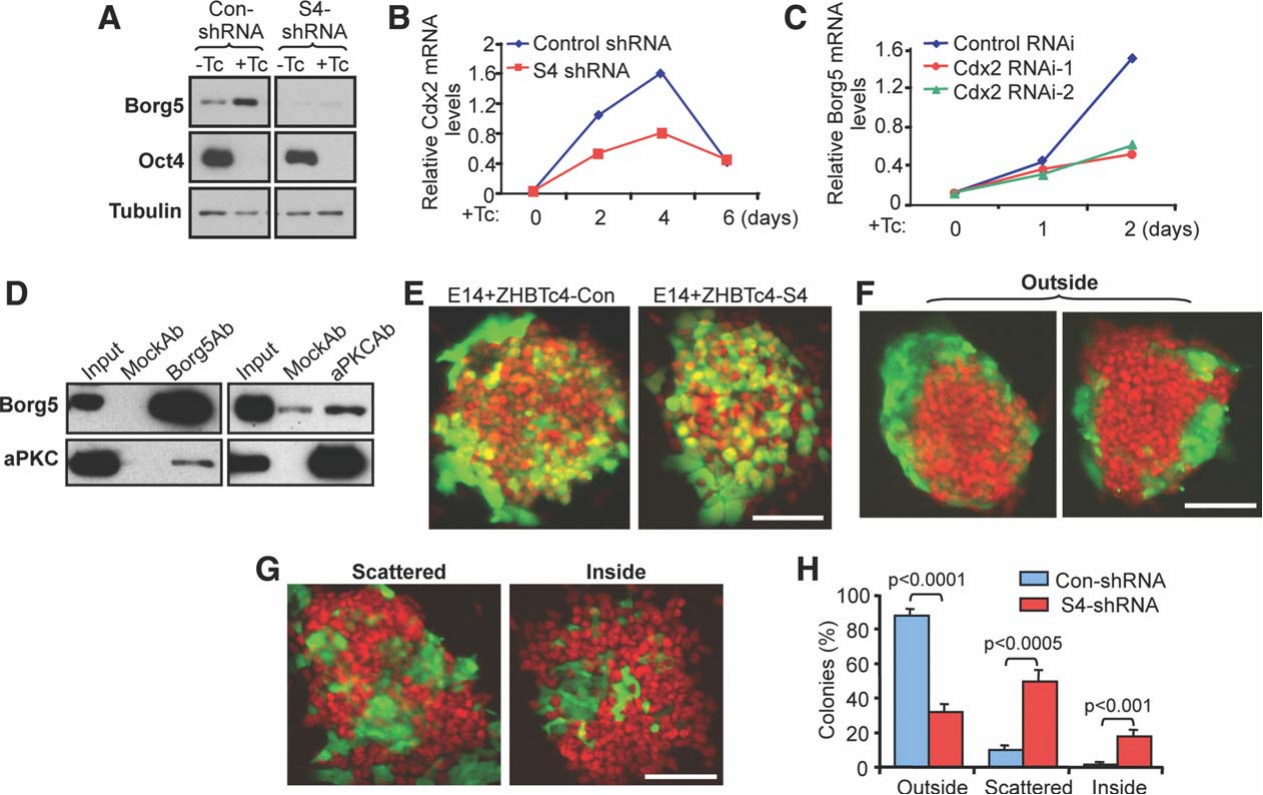
Borg5 regulates Cdx2 expression and trophectoderm (TE) cell sorting. (A): ZHBTc4-Con and ZHBTc4-S4 cells both downregulate Oct3/4 after Tc addition, but only ZHBTc4-Con cells upregulated Borg5 after Tc addition as judged by Western blotting. (B): Reduction of Borg5 by S4-shRNA caused reduction of *Cdx2* mRNA expression in ZHBTc4 cells after Tc addition. (C): Reduction of *Cdx2* expression by two different small interfering RNA oligos resulted in a reduction of *Borg5* mRNA expression in ZHBTc4 cells after Tc addition. (D): Borg5 interacts with aPKC in differentiating TE cells. Antibodies (Ab) to Borg5, aPKC, or control IgG (MockAb) were used for reciprocal immunoprecipitations in lysates made from the differentiating TE cells. (E): Images of E14 ESCs mixed with either ZHBTc4-Con or ZHBTc4-S4 ESCs expressing GFP (green). Oct3/4 antibodies (red) stained pluripotent ESCs. (F): Examples of colonies with TE cells repositioned to the outside of the colonies 42–48 hours after Tc addition. The differentiating ZHBTc4 cells are labeled green with GFP whereas the pluripotent E14 ESCs are labeled red by Oct3/4. (G): Examples of a scattered cell colony in which the differentiating green ZHBTc4 cells are scattered throughout the E14 ESCs (red) and an inside cell colony with the differentiating green ZHBTc4 cells enclosed inside the E14 ESCs (red). Scale bars in (E–G), 100 μm. (H): Quantification of colonies that have TE cells outside, scattered, or inside. Error bars, SEM. *p* values were calculated by Student's *t* test. Abbreviations: aPKC, atypical protein kinase C; RNAi, RNA interference; shRNA, short-hairpin RNA; Tc, tetracycline.

Since *Borg5* is upregulated during TE differentiation as early as *Cdx2*, we asked whether it could influence *Cdx2* expression. We created ZHBTc4 ESC lines that stably express either S4 short-hairpin RNA (shRNA) or control shRNA (Supporting Information). Both cell lines expressed Oct3/4 and were responsive to Tc, but ZHBTc4-S4 cells failed to upregulate *Borg5* as expected (Fig. 4A). qRT-PCR analyses showed that inhibiting *Borg5* during TE differentiation caused a corresponding reduction of *Cdx2* mRNA expression in these cells (Fig. 4B). Interestingly, reduction of *Cdx2* expression using two siRNA oligos targeting different regions of *Cdx2* also caused a corresponding reduction of *Borg5* mRNA expression (Fig. 4C).

**Figure 5:**
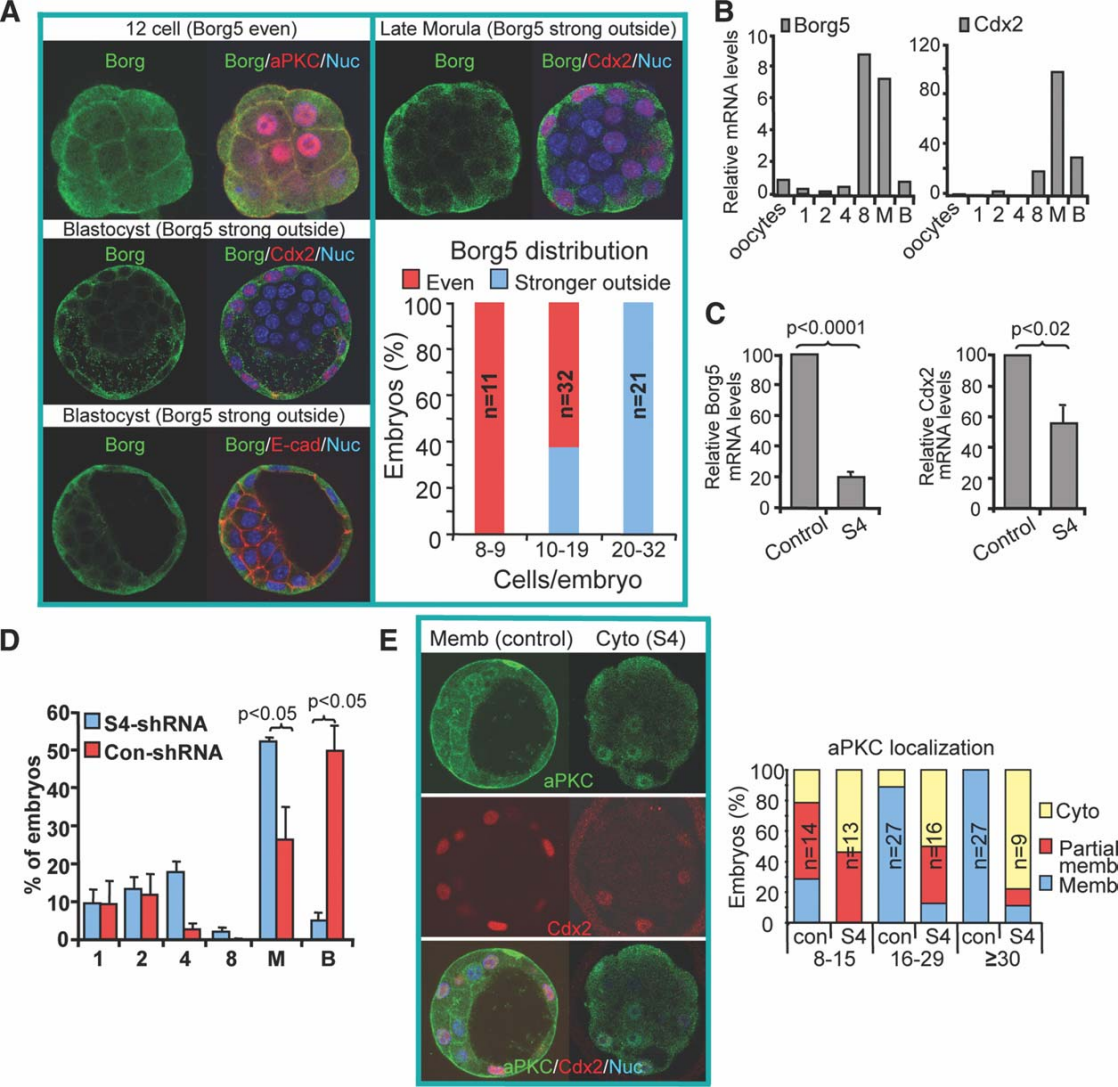
Borg5 promotes blastocyst formation. (A): Localization of Borg5 in the preimplantation embryos. Embryos from different stages were stained using antibodies to Borg5, aPKC, E-cadherin (E-cad), and Cdx2 as indicated. Nuclei were stained by DAPI. Borg5 localized to the cell–cell borders and cytoplasm. Quantifications revealed that many late morula and early blastocyst embryos had more Borg5 in outer cells than in inner cells (stronger outside). The number of embryos quantified in each group is shown on the histogram. (B): Quantitative real-time polymerase chain reaction analyses of *Borg5* and *Cdx2* expression in preimplantation embryos. Both *Borg5* and *Cdx2* are upregulated from eight-cell onward. M and B stands for morula and blastocyst, respectively. (C):*Borg5* shRNA expression (S4-shRNA) reduced expression of *Borg5* and *Cdx2* mRNAs. Morula embryos were used. (D):*Borg5* shRNA expression (S4-shRNA) reduced the formation of blastocysts when compared with control shRNA expression (con-shRNA). One, 2, 4, 8, M, and B in (B, D) refer to one, two, four, eight-cell, Morula, and blastocyst stage embryos, respectively. (E): Effects of *Borg5* reduction on aPKC localization. aPKC typically exhibits both membrane (memb) and cytoplasmic localization in control shRNA-injected embryos. However, *Borg5* (S4) shRNA injection resulted in embryos with largely cytoplasmic localization of aPKC (cyto). Quantification of embryos having normal membrane (memb), partial membrane (partial memb), or no membrane localization (cyto) is shown on the right. The number of embryos quantified in each group is shown on the histogram. Error bars, SEM. *p* value were calculated using Student's *t* test. Abbreviations: aPKC, atypical protein kinase C; shRNA, short-hairpin RNA.

The above results suggest the existence of a mutual positive feedback between TE cell morphogenesis and transcriptional regulation. This coupling is unlikely through a direct interaction between Borg5 and Cdx2 since they do not colocalize. Previous studies suggested that the atypical protein kinase C (aPKC) plays a role in TE differentiation upstream of Cdx2 [8, 23]. Using reciprocal immunoprecipitations, we found that Borg5 and aPKC interact with each other (Fig. 4D). This suggests that Borg5 could influence Cdx2 expression indirectly through regulating cell polarization and/or movement during TE differentiation.

### Borg5 Is Required for the Sorting of Differentiating TE Cells to the Outside of ESCs

TE cells are polarized epithelia that occupy the outside position in the blastocyst to enclose the ICM. We designed a cell-sorting assay to test whether Borg5 could be involved in this process. E14 ESCs were mixed with either ZHBTc4 ESCs expressing control (ZHBTc4-Con) or S4 shRNA (ZHBTc4-S4) and Tc was added to initiate TE differentiation from the ZHBTc4 ESCs (Supporting Information). ZHBTc4-Con and ZHBTc4-S4 cells also express GFP, which serves as a marker for identifying these cells. After 42–48 hours of Tc addition, cells were fixed and stained with antibodies to GFP and Oct3/4.

In the absence of Tc, ZHBTc4-Con or ZHBTc4-S4 ESCs (revealed by GFP staining) intermingled with the E14 ESCs in colonies (Fig. 4E). However, after Tc addition, the ZHBTc4-Con cells sorted away from the Oct3/4 positive E14 ESCs and often localized to the outside of the closely packed E14 ESCs (Fig. 4F, outside). By contrast, although groups of ZHBTc4-S4 cells still sorted together, they often formed small-cell clusters that scattered throughout the E14 ESCs or inside the E14 ESCs (Fig. 4G, scattered or inside). The E14 ESCs in these colonies often appeared less compact compared with the E14 ESCs that were cultured with the ZHBTc4-Con cells. We quantified the colonies blind (Supporting Information) and found that reducing Borg5 caused a significant decrease of colonies with TE cells on the outside of the colonies (outside) and a corresponding significant increase in TE cells scattered or enclosed inside of cell colonies (Fig. 4H). P-cadherin is expressed in TE. Since differential expression of cadherins in TE and ESCs could lead to their sorting, we asked whether Borg5 might regulate TE cell sorting through P-cadherin. We found that by 48 hours of Oct3/4 downregulation, P-cadherin was slightly upregulated (Supporting Information Fig. 5S). However, Borg5 reduction by two siRNA oligos (Supporting Information S2 and S4) did not affect P-cadherin expression in these cells (Supporting Information Fig. S5). Since Borg5 interacts with aPKC (Fig. 4D), these findings suggest that Borg5 promotes the sorting of differentiating TE cells to the outside of the undifferentiated ESCs by regulating cell polarization and motility.

### Borg5 Promotes Blastocyst Development

The above study led us to test whether Borg5 regulates blastocyst formation during preimplantation development. Immunofluorescence staining using affinity purified Borg5 antibodies generated in chicken revealed that Borg5 is localized along the cell–cell borders and in the cytoplasm in the post compaction embryos (Fig. 5A). Preabsorption using purified Borg5 protein showed that the staining was specific to the Borg5 protein (data not shown). From the late morula to blastocyst stage, many embryos had a higher amount of Borg5 in the outer cells than in the inner cells (Fig. 5A).

We found that both *Borg5* and *Cdx2* mRNAs were upregulated during eight-cell to morula stage (Fig. 5B) [24]. We microinjected S4-shRNA or control-shRNA construct into the pronuclei of fertilized mouse eggs. The expression of GFP in the injected embryos indicates the expression of the shRNA (Supporting Information Fig. S6). We found that only about half of the injected embryos expressed GFP. Nevertheless, the GFP-negative embryos served as controls because they had been subjected to the same micromanipulation and in vitro culturing conditions. As expected, S4-shRNA expression caused a significant reduction of *Borg5* in the embryos (Fig. 5C). Reduction of *Borg5* also significantly reduced *Cdx2* expression (Fig. 5C) and blastocyst formation (Fig. 5D).

Previous studies suggest that cell polarity and cell repositioning are essential for first lineage segregation and formation of blastocyts [7–9, 25]. Since Borg5 interacts with aPKC in differentiating TE cells (Fig. 4D) and since aPKC has been shown to regulate cell polarity in preimplantation embryos [23], we asked whether *Borg5* could regulate cell polarization by regulating aPKC localization during preimplantation development. Reduction of Borg5 by shRNA caused a disruption of aPKC enrichment at the plasma membranes in the embryos (Fig. 5E). Thus, Borg5 functions as an effector protein of Cdc42 to facilitate cell polarization and blastocyst formation by regulating aPKC localization.

## DISCUSSION

Most studies of ESCs have focused on transcriptional regulation during differentiation. However, organism development and stem cell differentiation involve gradual and coordinated morphological changes of individual cells in addition to changes of transcriptional programs. Identification of morphological regulators during differentiation could allow delineation of steps ESCs take to differentiate. By analyzing cell morphogenesis and migration during ESC differentiation, we show that distinct cell behaviors accompany differentiation of ESCs toward different lineages. The finding that early lineage differentiation of ESCs is accompanied by unique cell behaviors has allowed us to identify a cell morphological regulator Borg5 that regulates cell motility and sorting of differentiating TE cells.

Our studies show that Borg5 can enhance the expression of the lineage specific transcription factor Cdx2 and vice versa. As a transcription factor, Cdx2 could either directly or indirectly enhance *Borg5* gene expression. On the other hand, as a cytoplasmic protein, Borg5 most likely influence Cdx2 expression indirectly. Our cell sorting assay shows that Borg5 facilitates the positioning of differentiating TE cells to the outside of ESCs. Recent studies have revealed that cell polarity or cell–cell contacts could affect Cdx2 expression in preimplantation embryos [26]. It is possible that Borg5 could indirectly regulate Cdx2 expression by promoting the epithelial cell morphology in TE cells during differentiation. Consistent with this idea, our studies of Borg5 in preimplantation embryos have shown that Borg5 promotes blastocysts formation. Since Borg5 interacts with aPKC and it facilitates proper apical localization of aPKC in preimplantation embryos, we suggest that Borg5 regulates early TE differentiation by promoting TE polarization. This would in turn lead to further epithelial development in the TE cells.

Our studies show that ESCs can be used to identify morphological regulators that couple cell morphogenesis with transcriptional regulation during differentiation. The study of Borg5 provides the first step to further understand this coupling. A detailed understanding of this coupling should shed light on the developmental process in vivo. It will also provide insights into the steps that ESCs take to differentiate in vitro, which would facilitate the development of better methods to guide and enrich for the differentiation of morphologically correct and fate restricted cells from ESCs for efficient tissue engraftment.

## CONCLUSION

One of the most challenging problems in development is how the intricate transcriptional changes are coupled to gradual cell morphogenesis, leading to the build of tissues and organs. Although it is well established that transcription factors regulate cell morphogenesis, whether regulators of cell morphogenesis in turn influence expression of lineage-specific transcription factors is unclear. The cytoskeleton re-organization as cells migrate and change shapes during development could affect chromatin organization through the nuclear lamina, which would affect gene regulation. This idea suggests that by studying early cell morphogenesis as the mouse embryonic stem cells (ESCs) differentiate toward different lineages, it is possible to uncover cell morphology regulators that influence transcriptional regulation and vice versa. By analyzing early ESCs differentiation live, we have shown that unique cell morphogenesis indeed accompanies specific lineage differentiation from ESCs. By focusing on the differentiation of the trophectoderm lineage from ESCs, we have identified Borg5 as a morphological regulator that undergoes a similar strong up-regulation to Cdx2, a key transcription factor for trophectoderm differentiation. Our studies strongly suggest that Borg5 and Cdx2 reciprocally regulate each other to couple epithelial morphogenesis to transcriptional changes during development. Further study how Borg5 and Cdx2 regulate each other should help to decipher the steps of cell differentiation and tissue morphogenesis.
