# Supplementary figures and images for: A Role for Borg5 During Trophectoderm Differentiation
Source: Stem Cells. 2010 Apr 15;28(6):1030–8. doi: 10.1002/stem.428 (PMC2957878; doi:10.1002/stem.428)

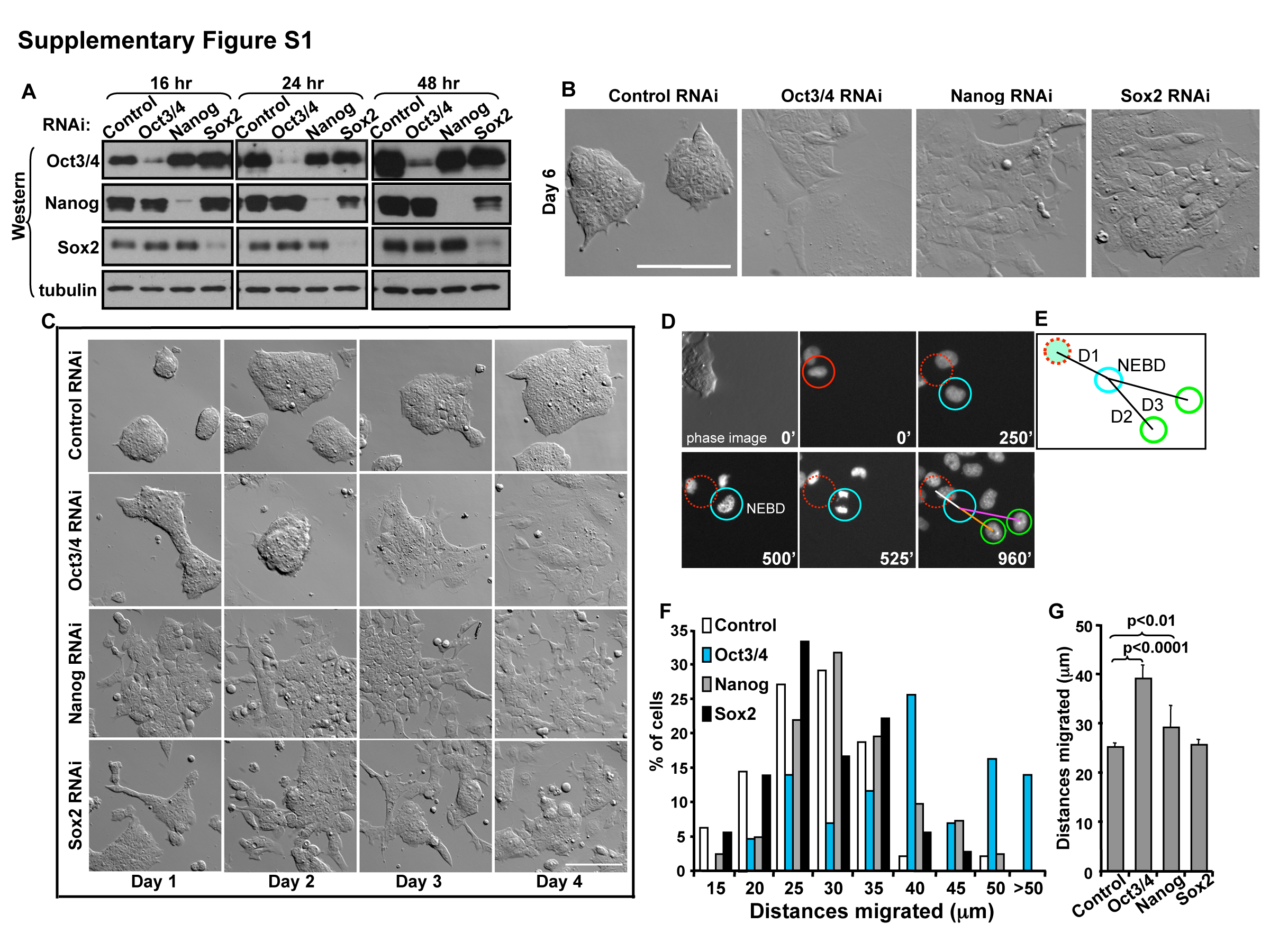

Supplement: Supplementary file 1 [file stem0028-1030-SD1.tif]

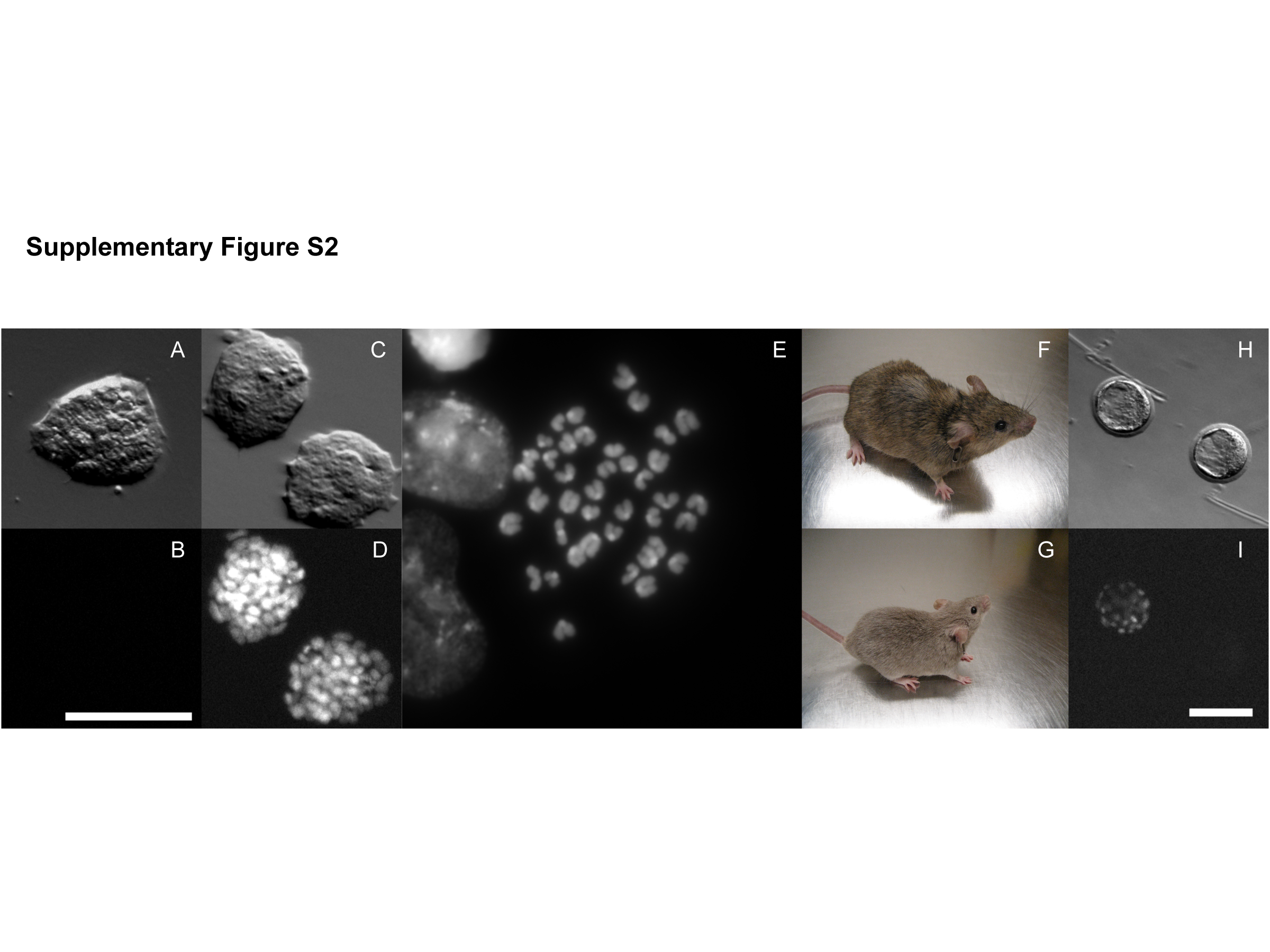

Supplement: Supplementary file 2 [file stem0028-1030-SD2.tif]

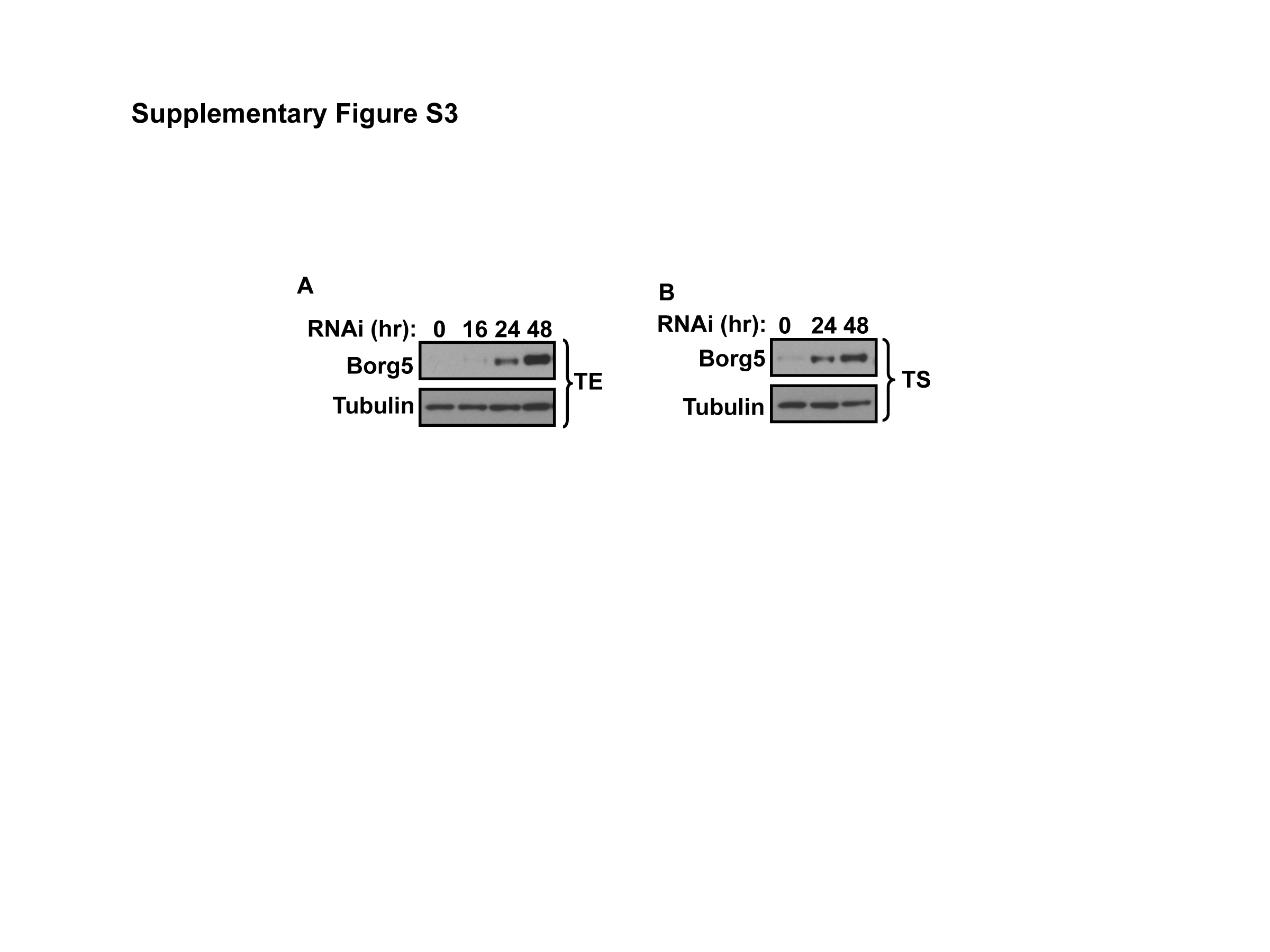

Supplement: Supplementary file 3 [file stem0028-1030-SD3.tif]

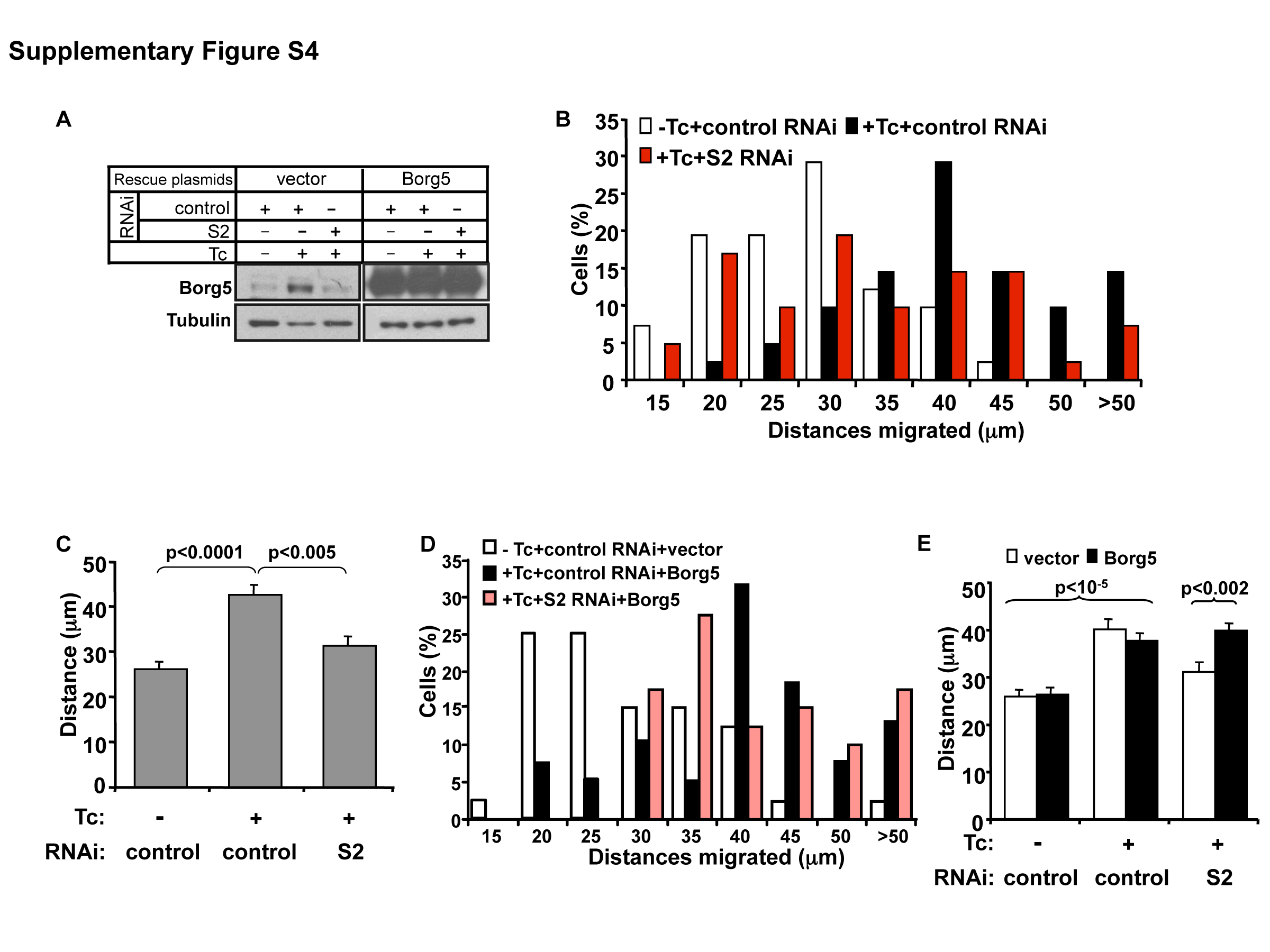

Supplement: Supplementary file 4 [file stem0028-1030-SD4.tif]

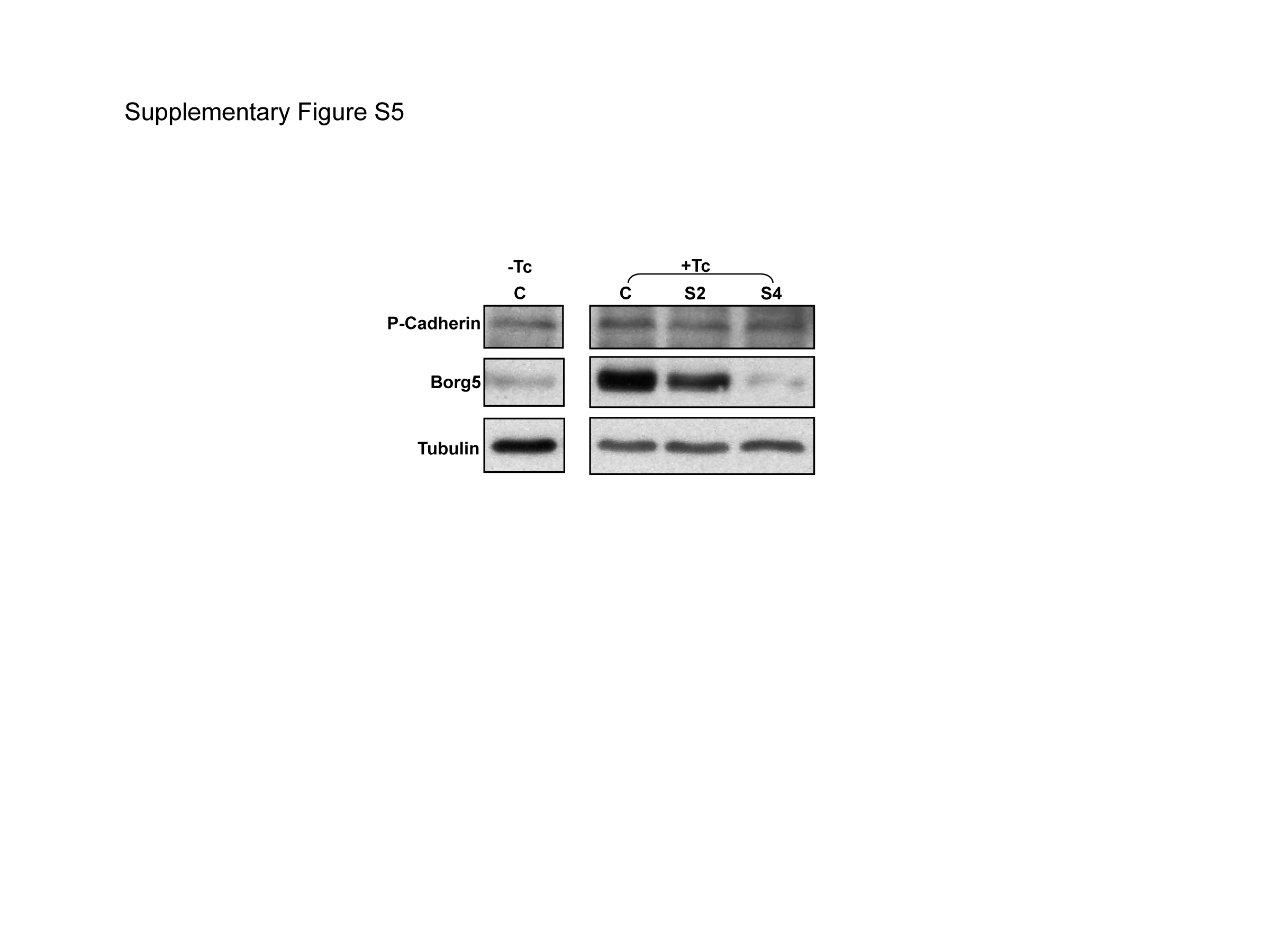

Supplement: Supplementary file 5 [file stem0028-1030-SD5.tif]

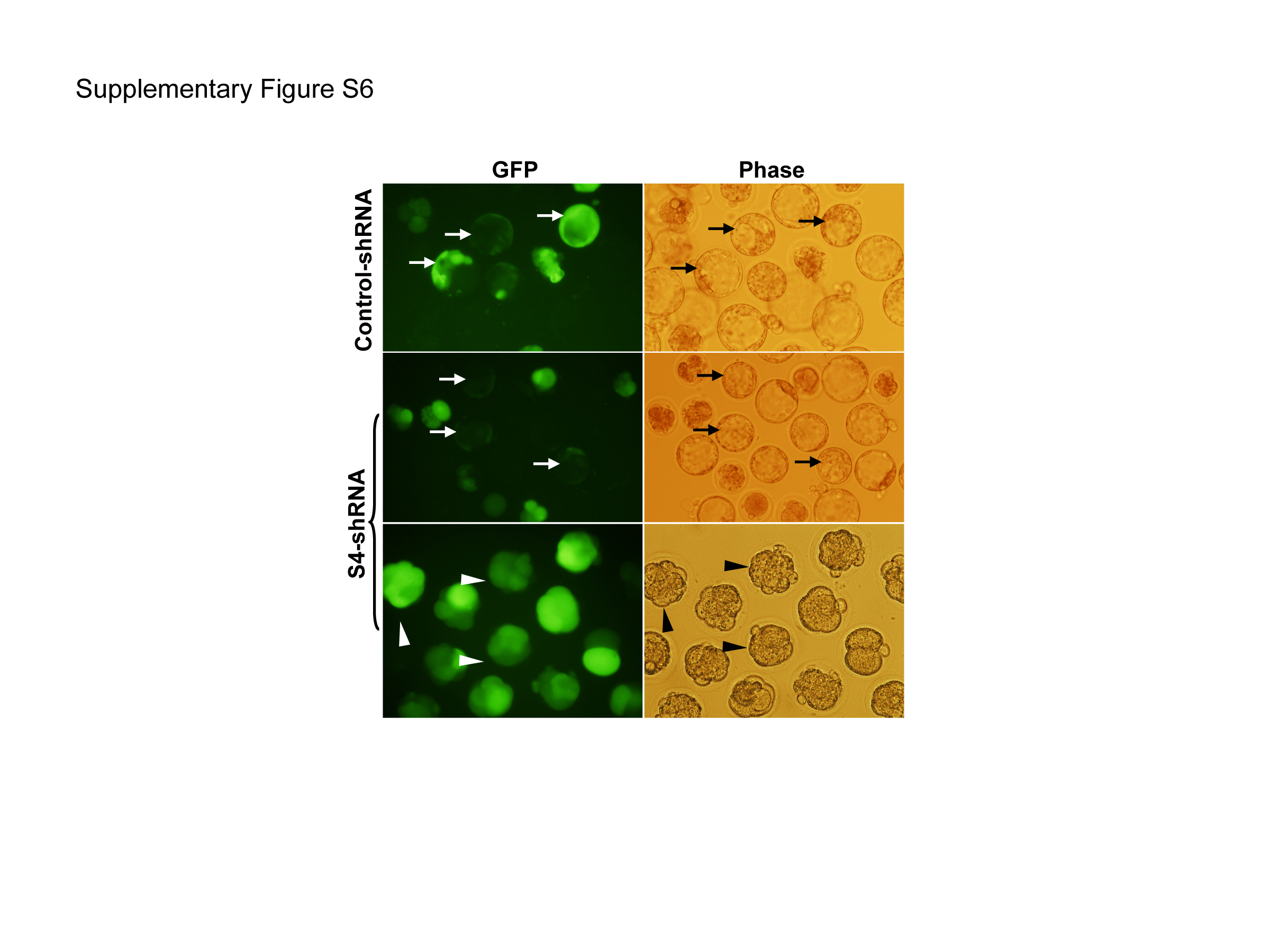

Supplement: Supplementary file 6 [file stem0028-1030-SD6.tif]

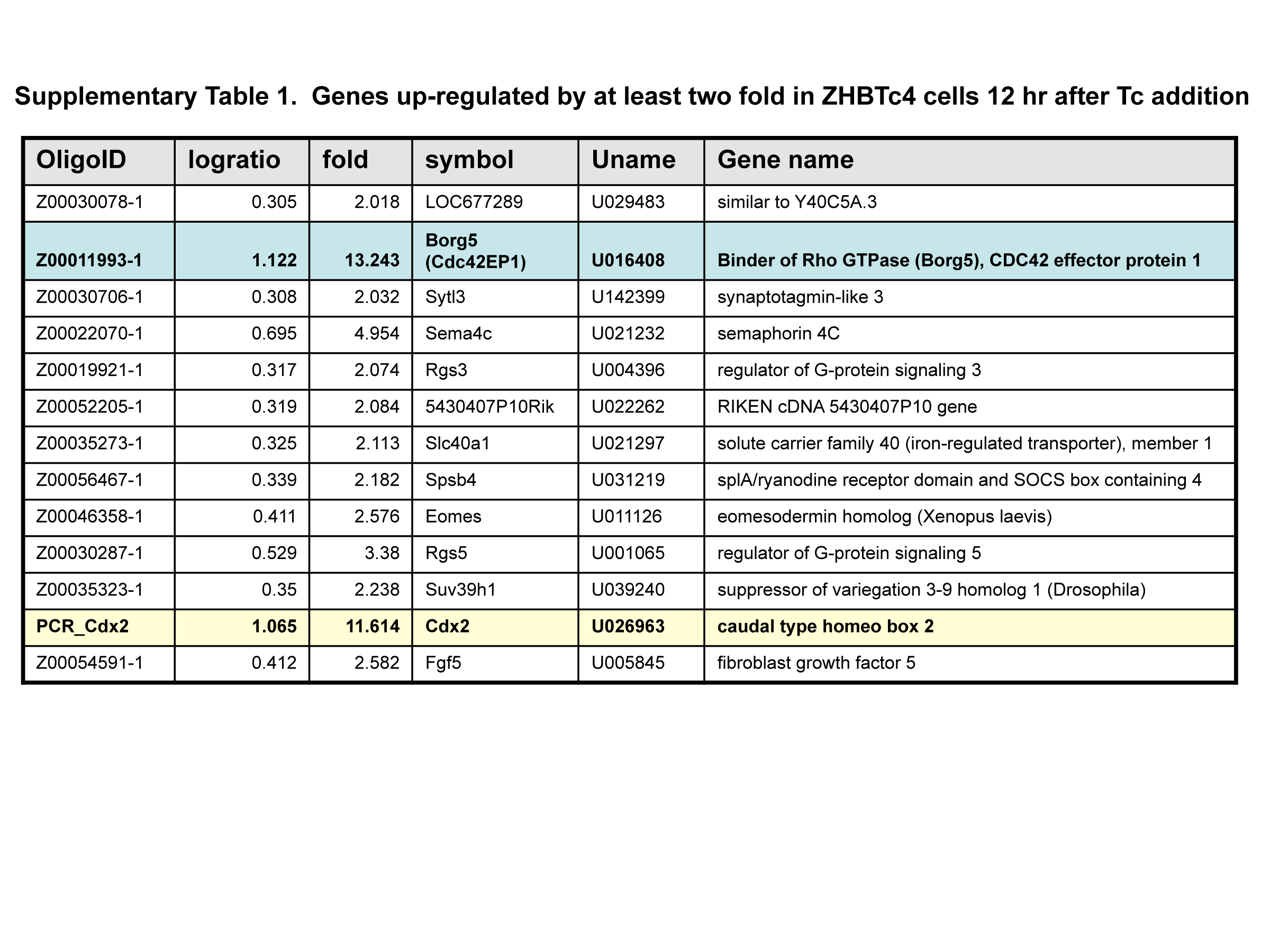

Supplement: Supplementary file 12 [file stem0028-1030-SD12.tif]
