# Supplementary material for: A Role for Borg5 During Trophectoderm Differentiation
Source: Stem Cells. 2010 Apr 15;28(6):1030–8. doi: 10.1002/stem.428 (PMC2957878; doi:10.1002/stem.428)
Supplement: Supplementary file 13 [file stem0028-1030-SD13.doc]

**A role for Borg5 during Trophectoderm Differentiation**

Queenie P. Vong, Zhonghua Liu, Jae Gyu Yoo, Rong Chen, Xie Wen, Alexei A. Sharov, Chen-ming Fan, Chengyu Liu, Minoru S. H. Ko, and Yixian Zheng

**Supplementary Information**

This supplementary information contains detailed materials and methods, 6 supplementary figures, 1 supplementary table, and 5 time-lapse movies.

**Plasmids, proteins, antibodies, and immunoprecipitations**

Plasmids expressing shRNA and GFP were constructed by modifying pLentiLox3.7 (pLL3.7) (http://www.sciencegateway.org/protocols/lentivirus/pllmap.html). The CMV promoter driving the expression of GFP in this plasmid was replaced by CAG promoter using the NotI and NheI sites. The primers for PCR the CAG promoter are: pCAG-NotI-F

taggga gcggccgc GTCGACATTGATTATTGACTAG, pCAG-NheI-R

tacccg gctagc CTCGAGGAATTCTTTGCCAAAATGATG. Control or S4-shRNA was cloned into the HpaI and XhoI sites according to <http://web.mit.edu/jacks-lab/protocols/pll37cloning.htm>. In the resulting construct the shRNA is expressed from the U6 promoter, while GFP is expressed from the CAG promoter.

Control shRNA sequences:

pLL3.7control-F

TcgtacgcggaatacttcgaTTCAAGAGATCGAAGTATTCCGCGTACGTTTTTTC

pLL3.7control-R

TCGAGAAAAAACGTACGCGGAATACTTCGATCTCTTGAATCGAAGTATTCCGCGTACGA

S4-shRNA sequences:

pLL3.7S4-F

TggaccattctcaagaccgaTTCAAGAGATCGGTCTTGAGAATGGTCCTTTTTTC

pLL3.7S4-R

TCGAGAAAAAAGGACCATTCTCAAGACCGATCTCTTGAATCGGTCTTGAGAATGGTCCA

Wild-type Cdc42, Cdc42Q61L (mimicking GTP-bound form), or Cdc42T17N (mimicking GDP-bound form) was tagged with FLAG at their N-terminus and cloned into pCAG-IP using XhoI and NotI sites. Wild-type or CRIB mutant Borg5 was cloned into pEF6/Myc (Invitrogen) using BamHI and NotI sites. To mutate the CRIB motif, the highly conserved amino acids (ISPPLG) were changed to AAPAAA using overlapping PCR-based mutagenesis. The primers for the overlapping PCR are as follows:

Borg5-CRIBmut-Forward primer:

**Gcc gct ccc gca gct gcg** gac ttc cgc cac acc atg cac g

Borg5-CRIBmut-Reverse primer:

Gtc **cgc agc tgc ggg agc ggc** cat gtc tgc tgt cag gcg tct c

The bold nucleotides correspond to the codons for the mutated amino acids.

To express the wild-type Borg5 or S2 and S4 RNAi-insensitive Borg5, wild-type Borg5 cDNA lacking the 5’-untranslated region or the same cDNA but harboring five mutations in the wobble codon correspond to the region of S4 was cloned into the pEF6/Myc (Invitrogen) in the BamHI and NotI sites. The CRIB motif mutated Borg5 was made into a S4 RNAi insensitive version.

The original Borg5 sequence: ccg gac cat tct caa gac cga.

The wobble codon mutated Borg5 sequence (capitalized letters were mutated wobble codons), which does not change the encoded amino acids: ccg gaT caC tcA caG gaT cga.

To make antibodies to mouse Oct3/4 and Sox2, the N-terminal Oct3/4 (aa, 29-124) was cloned into the EcoRI and XhoI sites in pGEX6p. The N-terminal Sox2 (aa, 1-198) was cloned into the BamHI and XhoI sites of pET30a. The GST-Oct3/4N and 6His-Sox2N proteins were expressed in bacteria (BL21) and purified for antibody production. The Sox2 antibody was purified against the 6His-Sox2N. To purify the Oct3/4 antibody, the antibody was first passed through GST-column to deplete the antibody against GST and then affinity-purified using GST-Oct3/4N.

To make antibodies to mouse Borg5 (409 aa), the C-terminal 100 aa (310-409) was cloned into BamHI and NotI sites in pGEX6p. The GST-Borg5C was expressed in BL21 and purified. GST-Borg5C was used for antibody production in chickens and rabbits, while the cleaved Borg5C protein devoid of GST was used to affinity-purify the antibodies.

The following antibodies were purchased. Primary antibodies: Nanog polyclonal Ab (Bethyl Laboratories), tubulin monoclonal Ab (DM1α, Sigma), Oct3/4 monoclonal Ab (C-10, Santa Cruz), Cdx2 monoclonal Ab (Cdx2-88, BioGenex), aPKC polyclonal Ab (aPKCzeta, C-20, Santa Cruz), aPKC monoclonal Ab (aPKCzeta, H-1, Santa Cruz), Cdc42 polyclonal Ab (P-1, Santa Cruz), goat anti-GFP Ab (Abcam), Secondary antibodies: donkey anti-goat (Alexa488, Molecular probes), goat-anti-rabbit (Alexa-488, Molecular Probes), goat-anti-mouse (Alexa-594, Molecular Probes), goat-anti-chicken (Alexa-546, Molecular Probes), goat-anti-chicken (Alexa-647, Molecular probes), goat-anti-rabbit (Alexa-647, Molecular probes), and goat-anti-mouse (Alexa-647, Molecular probes).

To study whether Borg5 interacts with Cdc42, HEK293T cells (~2 X 10^6^ cells per well of 6-well dish) were co-transfected with 2μg of FLAG-Cdc42 and 2μg of Borg5 or the respective vector controls using Lipofectamine 2000 (Invitrogen). Cells were incubated at 37°C for 48hr, then treated by lysis buffer (50mM Tris, pH 8.0 with 150mM NaCl, 1mM EDTA, 1% Triton X-100, and 1X protease inhibitor cocktail (Pierce)). Clarified cell lysates were incubated with 5μl of Borg5 antibody (rabbit) bound to protein G agarose (Invitrogen) or with anti-FLAG M2 affinity resin (Sigma), then agitated gently at 4°C overnight. After washes, the immunoprecipitates were analyzed by Western blotting probing with chicken Borg5 antibody (1:2000), rabbit Cdc42 antibody, or mouse FLAG M2 antibody (1:500, Sigma).

To study whether Borg5 interacts with aPKC, ZHBTc4 cells were plated on 10 cm dish coated with gelatin. Next day, cells were fed with fresh ESC medium with 1 μg/ml Tc. 48 hrs later, cells were treated with lysis buffer (25 mM Tris, pH 7.4, 150 mM NaCl, 0.5% Triton X-100, 1 mM EDTA) supplemented with protease inhibitor cocktail (Roche) and 2 mM Sodium Orthovanadate. Borg5 or aPKC (PKCzeta, Santa Cruz, H-1) antibodies were used for immunoprecipitation.

To study whether endogenous Borg5 in the differentiating TE cells interacts with Cdc42, ZHBTc4 cells were transfected with plasmid expressing FLAG-tagged wild-type Cdc42, Cdc42Q61L, or Cdc42T17N in suspension and allowed to attach to gelatinized plate overnight. Next day, cells were fed with fresh medium containing 1 μg/ml Tc and incubated for 2 days before immunoprecipitation. Cdc42 and Borg5 were pulled down by FLAG M2 agarose (Sigma) or antibodies against Borg5, respectively.

**ES cell cultures**

All ESCs were cultured in the absence of feeder cells (except when specifically indicated) in Glasgow Minimal Essential Medium (GMEM) supplemented with 10% fetal bovine serum (Hyclone), 1 mM sodium pyruvate, 2mM L-Glutamine, 10^-4^ M 2-mercaptoethanol, 1x non-essential amino acids (Invitrogen) and 1000 U/ml of LIF (ESGRO; Chemicon, Temecula, CA) on gelatin (Chemicon)-coated dishes.

**Establishment of ESCs expressing H2B-GFP**

A DNA fragment containing the hEF-1α promoter, H2B-GFP fusion gene, and SV40 poly-A was amplified from BOS H2BGFP-N1 vector (1) and then cloned into HindIII site in PL451 (http://recombineering.ncifcrf.gov/Plasmid.asp) to create a plasmid that expresses H2B-GFP from the hEF-1α promoter. To establish E14-H2B-GFP cells, 6x10^5^ E14 ESCs (MMRRC Baygenomics) were transfected with the linearized plasmid using Lipofectamine 2000 (Invitrogen) and plated on a 35 mm dish. Next day, cells were fed with fresh medium and incubated for one more day before re-plating onto a 10 cm dish and selected with 250 μg/ml G418 for 6 days. Single clones were obtained by limited dilution in 96 well plates. To check whether E14-H2B-GFP cells were pluripotent, a GFP positive clone B2 was tested for chimera formation and germ line transmission by injecting into blastocysts. The chimera mice were mated to obtain mice expressing H2B-GFP.

To establish ZHBTc4-H2B-GFP cells, ZHBTc4 cells were transfected with the H2B-GFP construct using Lipofectamine 2000. Single clones were obtained by limited dilution. Positive colonies were then selected by the GFP signal. Individual colonies were expanded to further test their morphology, Oct3/4 expression, and karyotype.

**Deriving ZHBTc4 cells stably expressing control-shRNA or S4-shRNA**

To create ES cells stably expressing control-shRNA or S4-shRNA, ZHBTc4 cells were co-transfected with the plasmid expressing either control-shRNA or S4-shRNA and pCAG-IP (providing puromycin resistance gene) (2) using Lipofectamine 2000. Puromycin (1μg/ml) was added for 6-7 days. Single GFP positive clones were picked and expanded. They were further tested for the presence of Oct3/4 and the ability to down-regulate Oct3/4 and up-regulate Borg5 in response to tetracycline.

**siRNA transfection of ES cells**

ESCs were transfected with complexes consisting of 80 nM siRNA and Lipofectamine 2000. Just prior to transfection, ESCs were trypsinized, pelleted by centrifugation, and resuspended into single cells. ESCs were then combined with freshly prepared transfection complexes and plated.

The following siRNA oligos were used:

Control:

Stealth™ RNAi Negative Control Medium GC (Invitrogen)

Control RNAi (Dharmacon): CGUACGCGGAAUACUUCGA

Oct3/4 (Stealth^TM^, Invitrogen) (3):

U1-GCCUUGCAGCUCAGCCUUAAGAACA

U2-CCCGGAAGAGAAAGCGAACUAGCAU

Nanog (Stealth^TM^, Invitrogen):

N8-UCCCGAGAACUAUUCUUGCUUACAA

N0-GGAGGACUUUCUGCAGCCUUACGUA

Sox2 (Ambion)

Sox2-2-GGAGUUUAUUCGGAUUUGA

Sox2-3-GGAUAAGUACACGCUUCCC

Borg5 (Dharmacon)

S4-GGACCAUUCUCAAGACCGA

S2-GCAGUUGUAAGCAAUCAAA

**Differentiation of ESC toward trophectoderm (TE)**

To differentiate E14 or ZHBTc4 or their derivatives toward trophoblast stem cell (TS) lineage, ~2.5-3.5 X 10^5^ of E14 cells transfected with control or Oct3/4 RNAi, or 3 X 10^3^ to 1 X 10^5^ of ZHBTc4 cells treated with tetracycline (Tc) were cultured in 12-well dishes in RPMI supplemented with 20% fetal bovine serum (Hyclone), 1 mM sodium pyruvate, 2mM L-Glutamine, 10^-4^ M 2-mercaptoethanol, 1x non-essential amino acids (Invitrogen), in the presence of 1 μg/ml of heparin (Sigma), and 25 ng/ml of recombinant FGF4 (Sigma) in the presence of 70% (v/v) of the MEF-conditioned medium (4). To differentiate toward the terminally differentiated TE lineage, 3 X 10^3^ to 1 X 10^5^ ESCs were cultured in the ES cell medium with Tc in 6-well dishes.

**Live imaging analyses of differentiating ESCs**

For imaging E14 ESCs after RNAi transfection, one part of E14-H2B-GFP cells was mixed with three parts of E14 cells. A total of ~2.5-3.5 X 10^5^ cells were transfected using control, Oct3/4, Sox2, or Nanog RNAi and then plated on a 12-well dish in ES cell medium. 16 hr after incubation, the dish was placed in a LiveCell^TM^ Chamber (Pathology Devices), which maintains the temperature at 37^o^C, humidity at 70-80%, and CO_2_ at 5%.

For imaging ZHBTc4 cells, ~2.5-3.5 x 10^5^ cells (1 part of ZHBTc4-H2B-GFP to 3 parts of ZHBTc4) were directly plated on a 12-well dish in ES cell medium. 16 hr after plating, 1 μg/ml Tc (final) was added to the culture before image acquisition. For imaging ZHBTc4 cells that were treated with S2 or S4 siRNA to silence the Borg5 expression, the cells were first transfected with the oligo and then imaged as above. To rescue Borg5 expression, the cells were first transfected with S2 or S4 siRNA together with 1 μg of empty vector, vector expressing Borg5 that is sensitive to S4 siRNA, or vectors expressing S4-insensitive Borg5 or Borg5 with mutations in the CRIB motif using Lipofectamine2000. 16 hr after transfection, the cells were changed into fresh ES cell medium with or without Tc before imaging.

Time-lapse sequences in multiple positions per well were acquired on an inverted microscope (TE2000-U, Nikon) driven by IPLab4.0 every 5 min for ~40 hr. Closed loop feedback from a Z Encoder Probe (Prior Scientific) was used to control focus drifts. Nuclear displacement was measured manually as described in the main text and in the Supplementary Movie 5 using the Metamorph program. At least 3 independent experiments were performed for each condition and more than 30 nuclei were analyzed for each experiment. Student T test was used to calculate the p values.

**Quantification of actin protrusion**

Alexa546-labeled phalloidin was used to stain actin in ESCs and differentiating ESCs. The actin protrusion length was determined from the centroids of the nuclei at the periphery of cell colonies to the tips of the longest actin protrusions. At least 10 randomly selected colonies were used and over 100 peripherally localized cells were measured. The experiments were repeated three times and a representative quantification was shown.

**Cell sorting assay**

The cell-sorting assay was modified from the embryoid culture (5). E14, ZHBTc4-control-shRNA, ZHBTc4-S4-shRNA ESCs were cultured on the mitomycin C treated MEF feeder. Cells were then kept in feeder-free condition for one passage before setting up the hanging-drop experiment. The cells were dissociated to obtain a single cell suspension. Hanging-drops of mixed cells were prepared by suspending 2.5 X 10^3^ cells per ml (Z4:E14=3:1) in ES cell medium in 20 μl drops on the lid of a 100 mm Petri dish. After culturing the hanging drop overnight, the aggregated cell clumps were seeded in a 6-well dish, then treated with tetracycline 6 hr later. After 40-44 hr of incubation, the cells were fixed and stained with mouse anti-Oct3/4 and goat anti-GFP antibodies. To analyze the colonies, 50-100 individual colonies for each experimental condition were imaged. All images were printed and scrambled together and then sorted blindly by a different person according to the distribution of Oct3/4 and GFP positive cells in the colonies.

**Embryo collection, culture, immunostaining, and imaging**

Female mice were injected with pregnant mare serum gonadotropin (PMSG) and human chorionic gonadotropin (hCG) (7.5 IU each, Calbiochem) to induce superovulation and then paired overnight with fertile males of the same strain (CD1 or B6D2F1). 18–20 hr after hCG injection, the plugged females were used to collect zygotes from the oviducts. The cumulus cells were removed by treatment with hyaluronidase (0.1% w/v, 300 Umg^-1^) in M2 medium, washed three times in M2 medium and then cultured in synthetic oviductal medium enriched with potassium (KSOMaa, MR-121-D, Millipore) to obtain different stage pre-implantation embryos. Alternatively, different stage pre-implantation embryos (2-cell to blastocysts) were collected by flushing oviducts and uteri with M2 culture medium ((MR-015-D, Millipore). Embryos were cultured in 50 μl micro-drops of KSOMaa under mineral oil in a humidified atmosphere of 5% CO_2_ at 37°C.

For immunostaining, embryos were fixed in 4% paraformaldehyde in PBS for 10 min and then permeabilized with 0.5% Triton X-100 in PBS for 30 min at room temperature. Before primary antibody incubation, the embryos were blocked in PBS containing 0.1% Triton X-100, 10% Fetal Calf Serum for at least 1 hr at room temperature or overnight at 4°C. After blocking, the embryos were incubated with different combinations of the following primary antibodies: mouse monoclonal Cdx2 antibody (1:500 dilution), rabbit or chicken Borg5 polyclonal antibodies (1:1000 dilution), polyclonal aPKC antibody (1:200 dilution), and/or monoclonal E-cadherin antibody (1:500 dilution) overnight at 4°C. The embryos were then washed in blocking solution for 15 min at room temperature for 3 times. After incubating in goat-anti-mouse, goat-anti-rabbit, and/or goat-anti-chicken secondary antibodies and DAPI in blocking solution for 1 hr at room temperature, the embryos were washed again in blocking solution 3 times for 15 min each at room temperature. Finally, embryos were mounted with 50% Glycerol in PBS.

All images of embryos were acquired on a Leica SP5 confocal microscope.

**Pronuclear injection of fertilized embryos**

The plasmids expressing control or S4-shRNA were linearized by cutting with ScaI and purified by phenol/chloroform extraction followed by ethanol precipitation with 0.1 M (final) NaCl (Promega, Cat#V4221). The DNA was separately microinjected into the pronuclei of fertilized mouse eggs following standard procedures as described in (6).  Four- to six-week old B6D2F1 mice (Jackson Laboratory) were superovulated by injecting 5 IU PMSG and 5 IU hCG (Calbiochem). Fertilized eggs (1-cell embryos) were harvested from mated superovulated mice at 20 hr after hCG injection. Hyaluronidase was used to remove cumulus cells. Embryos were thoroughly washed and selected for good morphology for collection. The linearized DNA was microinjected at the concentration of 2 μg/ml in 5 mM Tris (pH7.4) and 0.2 mM EDTA buffer.  After injection, Embryos were cultured in KSOMaa medium in a humidified 37^o^C incubator with 5% CO_2_.  Three to four days later, the embryos were transferred to M2 medium and examined under a fluorescence stereo-microscope (Nikon SMZ1500). The GFP positive and negative embryos were sorted according to their stages of development. Eight and eleven sets of injections were carried out for control-shRNA and S4-shRNA, respectively. Over 300 embryos for either control or S4-shRNA injection were analyzed.

To analyze whether Borg5 reduction inhibited blastocyst formation, the GFP-negative embryos were used as background controls for the damage caused by injection procedure and in vitro culturing. Embryos were analyzed four days after injection. To determine the percentage of embryos at each stage after expressing shRNA, the GFP-positive S4-shRNA or control-shRNA injected embryos were normalized using the GFP-negative S4-shRNA or control-shRNA injected embryos, respectively. Student T test was used to calculate the p values.

To analyze whether reduction of Borg5 by S4-shRNA was effective and whether it resulted disruption of aPCK localization or reduction of Cdx2 positive nuclei, the GFP-positive embryos were collected three days after microinjection and fixed for immunofluorescence analyses. For qRT-PCR analyses, GTP-positive morula were frozen on dry ice and then stored at -80^o^C until further analyses.

**Quantitative real time PCR analyses**

Total RNA from cells was prepared using RNeasy® Plus Mini kit (Qiagen) according to manufacture’s recommendations. First-strand cDNA was synthesized

from 1.5 μg of total RNA in a 20 μl reaction using the iScript^TM^ cDNA synthesis Kit (Bio-Rad). Real-time PCR reaction was done with the iQ^TM^ SYBR® Green Supermix (Bio-Rad) using the OPTICON^TM^ DNA engine from MJ Research. The relative amount of target RNA was determined from the appropriate standard curve and divided by the amount of GAPDH mRNA for normalization.

Primer sequences for QPCR:

Pre-designed QuantiTect primer (QT01574349) for Borg5 was purchased from Qiagen.

Cdx2-F CCTGCGACAAGGGCTTGTTTAG (7)

Cdx2-R TCCCGACTTCCCTTCACCATAC (7)

Gapdh-F TCCCACTCTTCCACCTTCGATGC (7)

Gapdh-R GGGTCTGGGATGGAAATTGTGAGG (7)

**Supplementary Figure Legend**

**Figure S1.** Effects of down-regulating Oct3/4, Sox2, or Nanog on ESC morphology and motility. (A) Treatment of E14 ESCs with Oct3/4, Sox2, or Nanog siRNAs resulted in reduction of the respective proteins as judged by Western blotting. (B) Reduction of Oct3/4, Sox2, or Nanog resulted in the flattening of ESC colonies after 6 days. (C) Reduction of Oct3/4, Sox2, or Nanog resulted in different colony morphology in the first four days. (D) Nuclear displacement as a measure of cell motility. The first phase-contrast frame shows the ESC colony at the beginning of the time-lapse series. This colony contains two H2B-GFP-positive cells. One of the nuclei is marked using a red circle. This nucleus displaced to a new position marked by a blue circle and then underwent nuclear envelope break down (NEBD) and mitosis. After mitosis, the two daughter nuclei migrated to two new positions marked by solid green circles at the end of the time-lapse movie. We measured nuclear displacement at NEBD by the white line connecting the centroids of the nucleus before displacement (dashed red circle) and the NEBD nucleus after displacement (blue circle). The daughter nuclear displacements were measured by the orange and pink lines connecting the centroids of the NEBD nucleus and the two daughter nuclei (green circles). (E) Schematic representation of nuclear displacement measurements. The sum of the nuclear displacements D1, D2, and D3 was used to assess cell motility. (F) Histogram distribution of cell migration after down-regulating Oct3/4, Nanog, and Sox2. At least 30 nuclei were measured in each condition. One of the three representative migration assays is shown. (G) Significant increase in cell motility as compared to control RNAi treatment was observed after down-regulating Oct3/4 or Nanog. Scale bar, 100 μm. Error bar, standard error of the mean (SEM). P values were calculated using Student T-test.

**Figure S2.** E14 ESCs expressing H2B-GFP are pluripotent. (**A**-**D**) Parental cell line E14 (**A** and **B**) and one clone called B2 E14 cells stably expressing H2B-GFP (in **C** and **D**) in bright field (**A** and **C**) and green fluorescence channel (**B** and **D**). (**E**) Karyotyping of B2 with DAPI staining. (**F**) Male chimera of B2 (623) was produced when B2 cells were injected into C57BL/6 blastocyst. (**G**) One of the F1 offsprings of 623 was mated with a CD1 female, which produced some blastocyst embryos that expressed H2B-GFP (**H**, bright field; **I**, fluorescence channel). Scale bars, 100 μm.

**Figure S3.** Reduction of Oct3/4 by RNAi caused up-regulation of Borg5 protein in E14 ESCs. E14 ESCs were transfected by Oct3/4 RNAi oligo and then cultured in either ESC medium, favoring TE differentiation, in (**A**) or in TS medium, favoring TSC formation, (**B**). Western blotting analyses showed that Borg5 protein was up-regulated 24 hr after siRNA transfection in both conditions.

**Figure S4.** Reduction of Borg5 expression by siRNA oligo S2 targeting the 5’ non-coding region reduced cell motility. (**A**) Borg5 suppression and rescue in ZHBTc4 cells under different conditions as indicated. Expression of Borg5 cDNA rescued Borg5 expression in cells treated with Tc and S2 siRNA. (**B** and **C**) Reduction of Borg5 by S2 siRNA inhibited cell migration induced by Tc-mediated Oct3/4 shutdown. Shown are distribution of distances migrated for >30 cells in each condition (**B**) and average distances migrated (**C**). (**D** and **E**) Expressing Borg5 from its cDNA rescued cell migration induced by Tc-mediated Oct4 shutdown. Shown are distribution of distances migrated for >30 cells in each condition (**D**) and average distances migrated (**E**). Since elevated Borg5 expression in ESCs (-Tc) or in differentiating ESCs (+Tc) did not induce enhanced cell migration as compared to cells that did not have elevated Borg5 (vector controls), overexpressing Borg5 did not cause gain of function in cell migration. Therefore, the rescue effect observed strongly suggests that the cell migration defects caused by S2 oligo was not due to off-target effect of siRNA. All the migration assays were repeated three times. Representative results from one experiment are shown. Error bars, SEM. P values were calculated using Student T-test.

**Figure S5.** Borg5 does not regulate P-cadherin expression. ESCs cultured in the absence of Tc (-Tc) and treated by control oligo (C) has low levels of P-cadherin expression, which was slightly up-regulated by reduction of Oct3/4 (+Tc) at 48 hrs. However, reduction of Borg5 by two oligos (S2 or S4) did not affect the level of P-cadherin in the differentiating cells at 48 hours of Tc addition.

**Figure S6.** Expression of shRNA is marked by GFP in the pre-implantation embryos. Examples of embryos injected with either control shRNA or S4-shRNA are shown. GFP expressing blastocysts or morula are indicated by arrows or arrowheads, respectively.

**Supplementary Table S1.** Genes that were up-regulated by at least 2-fold 12 hr after Tc addition in ZHBTc4. Microarray analyses were carried out using ZHBTc4 ESCs at 3, 6, 12, and 24 hr after Tc addition. No genes were seen up-regulated at 3 and 6 hr after Tc addition *(8,* 9). This table lists all 13 genes that were up-regulated by at least two-fold 12 hr after Tc addition. The two highest up-regulated genes are highlighted in blue and yellow. One of them is Borg5, which is also called Cdc42 effector protein 1 (Cdc42EP1), while the other is Cdx2.

**Supplementary Movies**

**Movie 1.** A phase contrast movie of E14 ESC treated with control RNAi.

**Movie 2.** A phase contrast movie of E14 ESC treated with Oct3/4 RNAi. Red arrows point to the dynamic long cell processes.

**Movie 3.**  A phase contrast movie of E14 ESC treated with Nanog RNAi.

**Movie 4.** A phase contrast movie of E14 ESC treated with Sox2 RNAi. Red arrows point to a cell that underwent extensive displacement before and after cell division.

**Movie 5.** Measurements of nuclear displacement. The first phase contrast frame is followed by H2B-GFP imaging. The red circle indicates the cell tracked. The blue circle indicates the position this cell underwent nuclear envelope breakdown. Two red arrows track the two daughter nuclei. The final positions of the two nuclei are outlined in green. The sum of distances D1, D2, and D3 was used to assess cell migration.

**References cited:**

1. T. Kanda, K. F. Sullivan, G. M. Wahl, *Curr Biol* **8**, 377 (1998).

2. H. Niwa, S. Masui, I. Chambers, A. Smith, J. Miyazaki, *Mol Cell Biol* **22**, 1526 (2002).

3. S. R. Hough, I. Clements, P. J. Welch, K. A. Wiederholt, *Stem Cells* **24**, 1467 (2006).

4. J. Quinn, T. Kunath, J. Rossant, *Methods Mol Med* **121**, 125 (2006).

5. R. L. Carpenedo, C. Y. Sargent, T. McDevitt, *Stem Cells* **25**, 2224 (2007).

6. P. M. Wassarman, M. L. DePamphilis, *Methods Enzymol* **225**, 747 (1993).

7. N. Ivanova *et al.*, *Nature* **442**, 533 (2006).

8. M. G. Carter *et al.*, *Genome Biol* **6**, R61 (2005).

9. A. A. Sharov *et al.*, *BMC Genomics* **9**, 269 (2008).
